# The Effect of Bovine Viral Diarrhoea Virus Biotypes on Bovine Oocyte In Vitro

**DOI:** 10.1002/vms3.70216

**Published:** 2025-04-10

**Authors:** Amirmahdi Roshanzamir, Massoud Talebkhan Garoussi, Jalil Mehrzad

**Affiliations:** ^1^ Department of Theriogenology, Faculty of Veterinary Medicine University of Tehran Tehran Iran; ^2^ Section of Immunology, Department of Microbiology and Immunology, Faculty of Veterinary Medicine University of Tehran Tehran Iran

**Keywords:** bovine viral diarrhoea virus, cytopathic, gene expression, non‐cytopathic, oocyte, total antioxidant capacity (TAC)

## Abstract

Bovine viral diarrhoea virus (BVDV) is a significant pathogen in the global cattle population, with two biotypes: cytopathic (CP) and non‐cytopathic (NCP), differing in their effects on cell culture. This study aimed to examine the impact of BVDV on the expression of apoptosis‐related genes and total antioxidant capacity (TAC) in vitro. Oocytes were obtained from post‐slaughter bovine ovaries and infected with both BVDV biotypes in vitro. Gene expression levels of bcl 2, bax, caspase 3 and caspase 9 were assessed using reverse transcription‐quantitative PCR (RT‐qPCR). The results indicated significant differences in gene expression levels, with bcl 2 expression reduced in CP and NCP‐infected oocytes compared to the control group (*p* < 0.05). Additionally, bax and caspase 3 expression levels were significantly elevated in the CP BVDV groups (*p* < 0.05). In the NCP BVDV groups, the expression of bcl 2 decreased (*p* < 0.05), while caspase 9 expression increased by 3.588‐fold compared to the control group (*p* < 0.05). Furthermore, TAC levels in the CP groups were significantly lower than those in the control group (*p* < 0.05). These findings suggest that the CP biotype of BVDV markedly affects TAC and alters the expression of key apoptosis‐related genes, while the NCP biotype reduces bcl 2 expression and increases caspase 9 expression.

## Graphical abstract

Bovine viral diarrhoea virus (BVDV) is a significant pathogen with two biotypes, cytopathic (CP) and non‐cytopathic (NCP). The aims were to examine the impact of BVDV on the expression of genes related to apoptosis and total antioxidant capacity (TAC) in vitro. Oocytes were infected with two BVDV biotypes. For assessing the bcl 2, bax, caspase 3 and caspase 9 gene expression, RT‐qPCR was used. The results showed significant differences in gene expression levels, including bcl 2, bax, caspase 3 and caspase 9, in the CP BVDV groups compared to the control group. Only bcl 2 and caspase 9 expression levels significantly decreased in the NCP BVDV groups compared to the control group. In addition, the CP groups TAC levels were much lower than those of the control group. It can be inferred that CP BVDV has a notable impact on the TAC of oocytes and alters the expression of genes such as bcl 2, bax, caspase 3 and caspase 9. However, the NCP biotype only leads to a decrease in expression of bcl 2 and caspase 9.

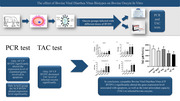

## 1 Introduction

One of the globally significant pathogens affecting cattle populations is the bovine viral diarrhoea virus (BVDV). This virus has attained enzootic status in terms of its wide distribution, absence of effective treatment and ease of transmission, posing a severe threat to dairy herds (Yeşilbağ et al. 2017). BVDV is a small, enveloped, single‐stranded, positive‐sense RNA virus with a genome size of about 12.5 kb. It belongs to the genus *Pestivirus*, family Flaviviridae. It possesses two biotypes: cytopathogenic (CP) and non‐cytopathogenic (NCP). The CP biotype causes apoptosis in cultured cells, whereas the NCP biotype does not (Dabiri et al. 2021). While the NCP biotype of BVDV is more prevalent and linked to severe acute infections, the less common CP biotype is primarily responsible for mucosal disease outbreaks (Oguejiofor et al. 2019). BVDV mainly infects cattle via the nasal route, although transmission can occur via semen and embryo transfer (Givens et al. 2009). The primary ramifications of contracting this virus are its detrimental effects on the foetus and its ability to weaken the immune system (Pinior et al. 2017). Subfertility is one of the significant economic losses associated with BVDV infection, in addition to abortion and foetal deformities during the later stages of the gestation period (Lanyon et al. 2014). Uterine endometrium is an important site for BVDV infection (Cheng et al. 2017). Viral infection can disrupt ovarian cycles, reduce fertility, cause repeat breeder syndrome and alter ovarian steroidogenesis (Wathes et al. 2020). Moreover, BVDV infection can inhibit folliculogenesis and reduce ovulation responses to superovulation protocols (Altamiranda et al. 2013; Oguejiofor et al. 2019). Ovarian granulosa cell necrosis, which is induced by CP BVDV infection, results in decreased estradiol production in the ovaries. Additionally, data suggest that necrosis is present in the oocytes of NCP BVDV‐infected calves (Seong, Oem, and Choi 2013). Furthermore, endometrial prostaglandin synthesis from prostaglandin F_2_ɑ to PGE_2_ was seen to be shifted by BVDV, rendering the uterus vulnerable to bacterial infections (Cheng et al. 2016; Fray et al. 2002). Reproductive failure and foetal loss associated with BVDV infection can be effectively mitigated through vaccination with either a modified live virus (MLV) or inactivated virus vaccine (IVV). Globally, these vaccines are implemented extensively (Perry et al. 2016).

The role of oxidative stress in viral infections has garnered considerable interest, especially given the critical involvement of reactive oxygen species (ROS) in the replication and disease development of various viruses (Aslan, Goksu, and Apaydin 2017; Sun et al. 2013). There is a dynamic balance between generating and eliminating free radicals in cells under normal circumstances. Nonetheless, this equilibrium may be disturbed when the production of free radicals exceeds the capacity of the systemic antioxidant defence mechanisms or when the antioxidant system is insufficiently effective in neutralising these free radicals (Bissoyi et al. 2014). Oxidative stress is a kind of disruption on the oxidant side that contributes to the development of many illnesses and inflammatory situations. Oxidative stress may cause cellular death by upregulating the expression of genes involved in apoptosis, such as bak 1, bax and caspase 3 while downregulating the expression of bcl2 (Efe et al. 2021). Apoptosis is a multifaceted physiological and pathological phenomenon intricately controlled by molecular pathways. The extrinsic and intrinsic pathways are particularly noteworthy as they play a crucial role in orchestrating cellular death (Lossi 2022). The extrinsic pathway includes the initiation of signalling cascades via interactions between receptors and ligands. More precisely, the process of attaching death ligands to death receptors situated on the outer surface of cells initiates the activation of initiator caspase 8. In contrast, the intrinsic route is regulated by a sequence of intracellular mechanisms that culminate in the release of cytochrome c from the mitochondria, subsequently triggering the activation of caspase 9 (Bissoyi et al. 2014). The aforementioned mechanisms ultimately intersect in a shared pathway that entails the proteolytic activation of caspases 3 and/or ‐7 from their inactive procaspase forms (Pietkiewicz et al. 2017).

Some viruses cause infected cells to undergo apoptosis (Hilbe et al. 2013). The buildup of double‐stranded RNA (dsRNA) in infected cells, which happens during RNA virus replication, is one element that initiates apoptosis via the intrinsic route (Price et al. 2022). The dsRNA‐dependent protein kinase (PKR) recognises dsRNA and starts kinase cascades that lead to apoptosis (Zuo et al. 2022). Furthermore, the CP biotype of BVDV can induce apoptosis through both intrinsic and extrinsic mechanisms by increasing the levels of dsRNA and producing tumour necrosis factor‐alpha (TNF‐α; Yamane et al. 2005). This study aimed to investigate the effect of the BVD virus on apoptotic and anti‐apoptotic gene expression levels and TAC in Holstein dairy cow oocytes.

## 2 Material and Methods

Bovine ovaries were acquired from a commercial slaughterhouse in the suburban areas of Tehran and Alborz provinces immediately after slaughter, and only one ovary from each mature cattle was submerged in phosphate‐buffered saline (PBS). Ovaries were transferred to the laboratory in 1 h and subjected to two more washes in fresh PBS containing streptomycin at a concentration of 100 mg/mL, penicillin at a concentration of 100 IU/mL and fungizone at a concentration of 25 µg/mL. Cumulus–oocyte complexes (COCs) were aspirated from ovarian follicles measuring 3–6 mm in diameter using an 18 gauge needle connected to a 10 mL syringe. Before starting any experimental procedures, follicular fluid extracted from each batch of ovaries was regularly tested using the Garoussi and Mehrzad (Garoussi, Mehrzad, and Nejati 2019) methodology for the presence of BVDV antigen using polymerase chain reaction (PCR).

### 2.1 Washing Oocytes

Before the in vitro maturation (IVM) of oocytes, they were subjected to a washing procedure based on the Stringfellow method (Stringfellow et al. 1997). This method involved ten cycles of washing. Initially, the oocytes were grouped into sets of 10 and placed within 1‐mL droplets of a washing medium. Subsequently, they underwent a single wash in an antiseptic solution for 90 s. Per the protocol, the washing medium contains 100 mL of PBS, 4 g of bovine serum albumin (BSA), 10,000 IU of penicillin G potassium and 10 mg of streptomycin sulphate. Moreover, as per the protocol, the antiseptic solution contains 100 mL of Hank's balanced salt solution (HBSS), 0.4 mL of trypsin‐ethylenediaminetetraacetic acid (EDTA; 0.25%), phenol red, 10,000 IU of penicillin G potassium and 10 mg of streptomycin sulphate.

### 2.2 In Vitro Maturation

Using the established procedures outlined by Garoussi and Mehrzad in 2011, bovine oocytes were matured in vitro. Oocytes were immersed in a maturation media and repeatedly washed, surrounded by layers of dense follicular cells. Tissue culture medium‐199 (TCM‐199) supplemented with Earle's salts, 10% BVDV‐free foetal bovine serum (FBS), 1 µg/mL estradiol, 60 µg/mL Folltropin, 2 IU/mL human chorionic gonadotropin (HCG), 50 ng/mL epidermal growth factor (EGF), 0.4 mM glutamine, 0.2 mM sodium pyruvate and 50 µg/mL gentamicin made up the maturation medium. Four well‐culture plates containing 700 µL of the maturation medium were used to culture the oocytes in an incubator. The incubation conditions consisted of 38.5°C temperature, 5% carbon dioxide (CO_2_) concentration and 100% humidity for 24 h. After maturation, the cumulus cells were removed from the oocytes by vortexing them for 90 s in 2 mL of minimal essential medium (MEM) containing 2% BVDV‐free foetal calf serum (FCS).

### 2.3 Virus Culture and Preparation

The Madin–Darby bovine kidney (MDBK) cell line was used for the BVDV laboratory culture. The CP biotype of BVDV was obtained from the Department of Virology at the Faculty of Veterinary Medicine, University of Tehran, Tehran, Iran, while the NCP biotype was acquired from the Virology Department at the Razi Vaccine and Serum Research Institute, Iran. The BVD viruses used in this study were the CP strain Oregon C24 V and the NCP isolate No. 22146 (Garoussi 2007). Initially, MDBK cells were cultured in MEM supplemented with 5% FCS. Adherent cells were subcultured after reaching 70%–80% confluence. The virus was propagated in MDBK cells and frozen at −70°C. After eight passages, the presence of the virus was confirmed using the reverse transcription‐polymerase chain reaction (RT‐PCR). The virus was then frozen and titrated using the Reed–Muench formula (Reed and Muench 1938).

### 2.4 Experimental Groups

The experimental groups were established based on the CP and NCP biotypes of the BVD virus, with two different viral doses (10^5^ and 10^4^) expressed as 50% tissue culture infectious dose (TCID_50_/mL), as described by Garoussi and Mehrzad (2011).

The oocytes were divided into five distinct groups, each consisting of at least 60 oocytes, and replicated three times. The control group was not exposed to any BVDV biotypes. Twenty‐two hours after maturation, oocytes were infected with two doses of BVDV biotypes in the medium for 2 h at 38.5°C temperature. In brief, the second and third groups were infected with 10^4^ and 10^5^ TCID_50_/mL of NCP BVDV, respectively. The fourth and fifth groups were infected with 10^4^ and 10^5^ TCID_50_/mL of CP BVDV, respectively.

### 2.5 RNA Extraction and RT‐qPCR

Each group was subjected to RT‐qPCR analysis to assess viral infection's effect on gene expression. RNA was extracted following a standard protocol with the FavorPrep Tissue Total RNA Mini Kit. Samples were treated with 350 µL FARB buffer and 3.5 µL β‐mercaptoethanol. They were then allowed to incubate for 5 min at room temperature. The sample mixtures were then moved to filter columns, where they were centrifuged for 2 min at maximum speed (18,000 × *g*) to collect the cleared supernatants in fresh microcentrifuge tubes. An equal volume of 70% RNase‐free ethanol was added to collected supernatants to remove contaminants, followed by thorough mixing via vortexing. The resulting mixture was then transferred onto mini‐columns placed in collection tubes. After centrifuging at maximum speed for 1 min, the flow‐through was discarded, and the mini‐columns were repositioned in the collection tubes. Washing buffer (500 µL) was added to the mini‐columns, followed by another round of centrifugation at maximum speed for 1 min. The flow‐through was discarded, and the mini‐columns were returned to the collection tubes.

A second washing step was performed by adding 750 µL of washing buffer to the mini‐columns, followed by centrifugation at maximum speed for 1 min. The flow‐through was discarded, and the mini‐columns were again placed in the collection tubes. An additional centrifugation step was conducted for 3 min to dry the mini‐columns.

RNA was eluted by carefully placing the mini‐columns in elution tubes, adding 50 µL of RNase‐free ddH_2_O to the centre of the membrane, and allowing it to stand for 1 min. Subsequent centrifugation at maximum speed for 1 min facilitated the elution of RNA from the mini‐columns into the elution tubes.

The concentration of total RNA in each sample was determined by utilising a NanoDrop ND‐1000 UV–vis spectrophotometer (NanoDrop Technologies Inc.) to measure absorbance at 260 nm. The A260/A280 ratio was used to determine the RNA's purity. The Rotor‐Gene Q system (Qiagen, Valencia, CA) was used to conduct real‐time PCR for the relative quantification of mRNA copies. The YTA SYBR Green qPCR Kit (Yekta Tajhiz Azma, Tehran, Iran) was used following the manufacturer's guidelines. cDNA synthesis was carried out per the manufacturer's instructions before PCR.

### 2.6 Total Antioxidant Capacity Assay

The TAC test was used to assess the samples' overall antioxidant capacity with and without extract. This experiment's concept and methodology include the reduction of Fe^2+^ ions. The process produced a dye when the chromogen 2,2′‐azino‐bis(3‐ethylbenzothiazoline‐6‐sulphonic acid) was used as an appropriate substrate for the peroxidase enzyme. The absorbance of light at 593 nm was measured, and the optical density (OD) was recorded. A higher antioxidant power in the sample indicates the increased involvement of the chromogen in the reactions, resulting in higher OD values. An optimisation process was performed to quantify the antioxidant levels in the samples, and a standardised method was employed to report the results. Utilising a standard working solution, light absorption was measured. In order to conduct the assay, 5 µL of the sample were added to a 96‐well plate, while 5 µL of PBS were added to the wells serving as the control group. Afterwards, 250 µL of the working solution was added to each well. The plate was incubated for approximately 5 min at ambient temperature. The concentration of Fe^2+^, representing the antioxidant capacity and the presence or absence of antioxidants in the samples, was determined and reported as µM Fe^2+^/L.

### 2.7 Statistical Analysis

The results were analysed using GraphPad Prism version 8.4.3. One‐way analysis of variance (ANOVA) with post hoc Tukey's honestly significant difference (HSD) test was used to determine the statistical significance between groups. A *p* value of ≤0.05 was considered significant. The results were presented as means ± standard error of the mean (SEM).

## 3 Results

### 3.1 Analysis of Bcl 2 Expression

**FIGURE 1:**
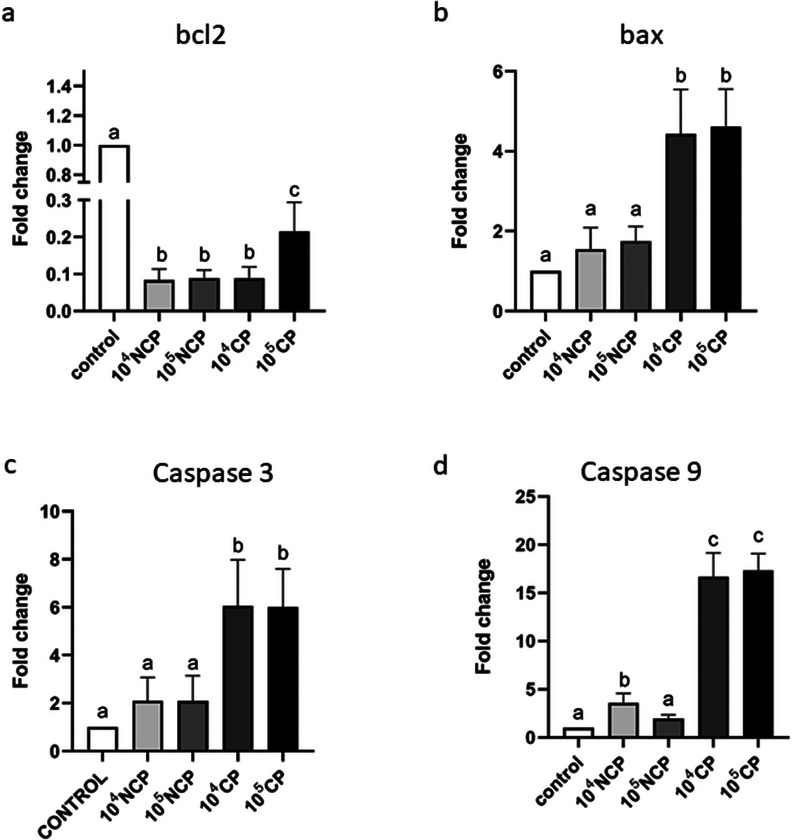
Presents gene expression analysis in bovine oocytes. When compared to the control group, all experimental groups treated with both the cytopathic (CP) and non‐cytopathic (NCP) biotypes of the bovine viral diarrhoea virus (BVDV) showed a significant decrease in the expression of the anti‐apoptotic bcl 2 gene (*p* < 0.05). When compared to the control group, the pro‐apoptotic bax gene expression was significantly higher in the experimental groups treated with the CP biotype of the BVDV (both dosages; *p* < 0.05). Only the CP biotype of the virus (both doses) significantly raised the expression of the caspase 3 gene (*p* < 0.05), according to the analysis of its expression. Compared to the control group, the expression level of the caspase 9 gene was significantly higher in the CP‐infected groups (*p* < 0.05). Furthermore, only 10^4^ NCP group significantly increased caspase 9 gene expression compared to the control group (*p* < 0.05). However, the 10^5^ NCP group did not increase this gene expression compared to the control group (*p* > 0.05). The graph's columns with distinct alphabetic letters have substantially varied ^a, b, c^ and ^d^ symbols.

Both biotypes of BVDV had a significant effect on bcl 2 gene expression (*p* < 0.05; Figure 1a). The expression level of the bcl 2 gene was significantly lower in all experimental groups compared to the control group (*p* < 0.0001).

### 3.2 Analysis of Bax Gene Expression

The CP biotype of the BVDV significantly affected the bax gene expression of infected oocytes (*p* < 0.05; Figure 1b). Compared to the control group, the 10^4^ TCID_50_/mL NCP and 10^5^ TCID_50_/mL NCP groups, and the control group, both doses of the CP biotype significantly increased bax gene expression (*p* < 0.0001). Conversely, the gene expression level of the 10^4^ TCID_50_/mL NCP and 10^5^ TCID_50_/mL NCP groups was marginally higher than that of the control group, and their actual fold changes were 1.544 and 1.746; However, this difference was not statistically significant (*p* > 0.05). When compared to the control group, the *p* values of NCP groups were (*p* = 0.3401) and (*p* = 0.1034), respectively.

### 3.3 Analysis of Caspase 3 Expression

The expression level of the caspase 3 gene was significantly higher in both CP experimental groups compared to the control group (*p* < 0.05; Figure 1c).

Caspase 3 expression was significantly higher in the 10^4^ TCID_50_/mL and 10^5^ TCID_50_/mL CP biotype groups compared to the control and other treatment groups (*p* < 0.0001). The fold changes in caspase 3 expression for the 10^4^ and 10^5^ TCID_50_/mL CP groups were 6.02 and 6.05, respectively, relative to the control group. The caspase 3 gene expression was not statistically significantly increased by the 10^4^ TCID_50_/mL and 10^5^ TCID50/mL concentrations of the NCP biotype compared to the control group (*p* > 0.05). The actual *p* values were (*p* = 0.2177) and (*p* = 0.2477).

### 3.4 Analysis of Caspase 9 Expression

The expression level of the caspase 9 gene was significantly higher in both doses of the CP and 10^4^ NCP biotype of the BVDV compared to the control group (*p* < 0.05; Figure 1d).

The caspase 9 gene expression was significantly higher in the 10^4^ TCID_50_/mL and 10^5^ TCID_50_/mL CP groups compared to the control and other treatment groups, with an actual fold change of 16.65 and 17.35, respectively (*p* < 0.0001). Furthermore, the 10^4^ NCP group has increased this gene expression significantly compared to the control group (*p* = 0.0004), and this group's fold change was 3.588. However, comparing the 10^5^ TCID_50_/mL NCP group to the control group did not reveal a statistically significant increase in caspase 9 gene expression (*p* = 0.4732).

### 3.5 Total Antioxidant Capacity Assay

**FIGURE 2:**
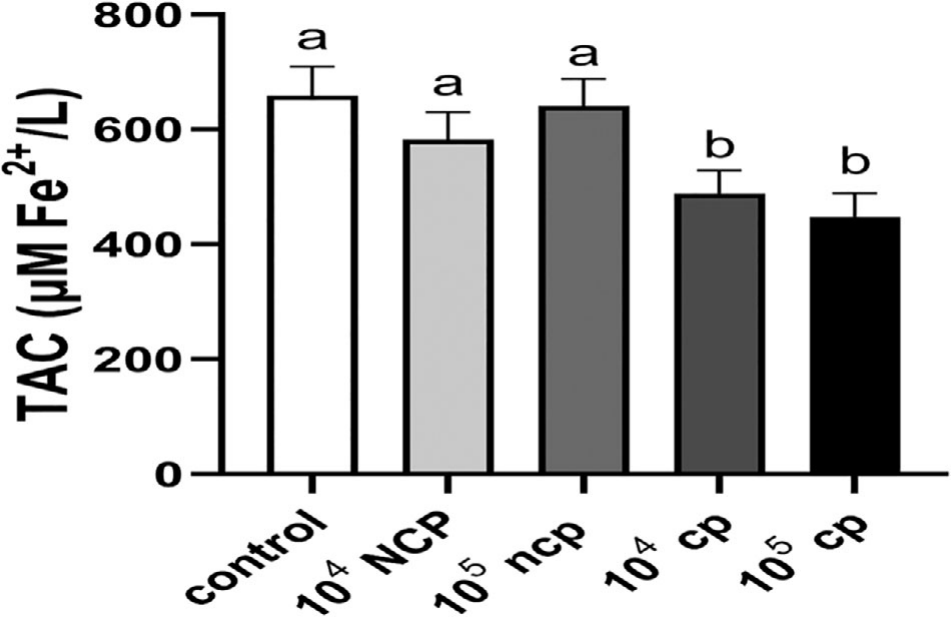
The total antioxidant capacity (TAC) of bovine oocytes is shown in this figure as a function of two dosages (10^4^, 10^5^ TCID_50_/mL) of the bovine viral diarrhoea virus (BVDV) biotypes that are cytopathic (CP) and non‐cytopathic (NCP). The results showed significant differences between the 10^4^ TCID_50_/mL and 10^5^ TCID_50_/mL dosages of CP biotype‐infected oocytes and the control group (*p* < 0.05). This suggests that the CP biotype infection has caused a significant drop in TAC levels. The control group and the various dosages of NCP biotype‐infected oocytes did not significantly vary from one another, suggesting that the TAC levels in these groups were comparable. Columns in the graph labelled with different alphabetic letters (^a, b^) are significantly different.

The effects of several BVDV biotypes on oocytes are shown in Figure 2. TAC for the control group was 659.3 ± 50.44. TAC levels were similar in the control group and the 10^4^ TCID_50_/mL and 10^5^ TCID_50_/mL NCP groups, with no significant differences (*p* > 0.05). However, there was a significant reduction in TAC when comparing the 10^4^ TCID_50_/mL and 10^5^ TCID_50_/mL CP groups to the control group after infecting the oocytes with different amounts of the cytopathic (CP) biotype of BVDV (*p* < 0.0001).

## 4 Discussion

Building on prior research showing that BVDV can induce apoptosis through intrinsic and extrinsic pathways and impact antioxidant capacity (Gao et al. 2022), this study examined how BVDV affects apoptotic gene expression and antioxidant capacity in infected bovine oocytes.

Both biotypes of the BVDV were found to downregulate the expression of the anti‐apoptotic bcl‐2 gene. Furthermore, the CP biotype demonstrated a substantial increase in apoptotic gene expression for bax and caspase 3 at dosages of 10^4^ and 10^5^. Nevertheless, the expression of these genes did not exhibit a substantial change in the NCP biotype. Additionally, when comparing caspase 9 gene expression between the treatment and control groups, the 10^4^ NCP, 10^4^ CP and 10^5^ CP groups revealed increased expression, while the 10^5^ NCP group did not exhibit a significant change in gene expression.

The TAC assay demonstrated that the virus's CP biotype significantly reduced the infected oocytes' antioxidant capacity. This reduction may be attributed to the CP biotype's CP effects, which likely lead to increased oxidative stress and depletion of antioxidant reserves in the oocytes. In contrast, the NCP biotype did not induce a statistically significant reduction in antioxidant capacity at either tested dose. This lack of significant impact could be due to the non‐cytopathic (NCP) nature of the NCP biotype, which may not exert the same level of oxidative stress or disrupt cellular antioxidant mechanisms. These findings suggest that the distinct pathogenic mechanisms of the two biotypes contribute to their varying effects on oocyte antioxidant capacity.

The elucidation of mechanisms underlying cell injury induced by BVDV is paramount to comprehend this disease's pathogenesis. In particular, the virus's CP biotype was associated with distinctive morphological alterations in infected host cells, culminating in apoptotic cell death (Abdelsalam et al. 2020).

Apoptosis pathways may be initiated by signals from either outside or within the cell, which finally come together to activate caspase 3, the critical executioner of caspase in apoptosis (Hilbe et al. 2013).

Cells infected with the CP biotype of BVDV show distinct morphological and structural alterations and the activation of specific genes associated with viral effects on the infected cells (Abdelsalam, Kaushik, and Chase 2023). Our research showed that the CP biotype of the virus can upregulate the expression of genes involved in the extracellular and intracellular pathways of apoptosis in bovine oocytes, which is in line with earlier findings by Grummer and Yamane (Grummer et al. 2002; Yamane et al. 2005). The caspase 3, 9 and bax gene's expression were significantly elevated in the virus's CP biotype. On the other hand, only the expression of the caspase 9 gene was significantly elevated in the NCP biotype. These differences in apoptosis induction may arise from the distinct interactions of the CP and NCP biotypes with host cells. The CP biotype, due to its CP nature, may more aggressively disrupt cellular homeostasis, leading to the activation of multiple apoptotic pathways. In contrast, the NCP biotype, being NCP, likely engages in less extensive interaction with apoptotic pathways, resulting in minimal or no significant activation of these pathways. This suggests that the CP biotype has a more potent pro‐apoptotic effect, contributing to more rapid and widespread cell death compared to the NCP biotype. The observed lower expression of apoptotic genes in NCP biotype‐infected oocytes may be attributed to the effect of this biotype on the infected oocytes, which does not induce apoptosis in these cells. This differential effect on apoptotic gene expression suggests that the NCP biotype may employ alternative strategies to modulate the cellular response, distinct from apoptosis, in infected oocytes, which correlates with Grummer, Yamane and Choi's findings (Choi 2017; Grummer et al. 2002; Yamane et al. 2005).

In cells infected with the CP biotype of BVDV, the start of viral replication is accompanied by an instantaneous breakdown of mitochondrial membrane potential (Grummer et al. 2002; Zhou et al. 2017). The degradation of mitochondrial membrane potential is a critical event in viral infection‐induced cellular alterations (Wang et al. 2021). Mitochondrial membrane potential is vital to maintaining cellular homeostasis, producing energy and regulating apoptotic pathways. The breakdown of mitochondrial membrane potential observed in CP BVDV‐infected cells suggests a disruption in mitochondrial function and cellular metabolism (Reuscher et al. 2021).

BAG, bcl 2, bcl x, bcl xl and other anti‐apoptotic members of the bcl 2 family work by binding to and blocking pro‐apoptotic proteins, which stops cytochrome c from being released (Bissoyi et al. 2014). Notably, low levels of bcl 2 expression were associated with increased susceptibility to apoptosis (Du et al. 2020). Consistent with these findings, the results of the present study showed a decrease in the expression level of the bcl 2 gene across all treatment groups, supporting the observations made by Pedrera et al. (2012).

Furthermore, while our results highlight the impact of BVDV on apoptosis and TAC of bovine oocytes, it is crucial to acknowledge that apoptosis is a complex process involving multiple pathways and factors beyond those examined in this study. We focused on bcl‐2, bax and caspase genes; however, this approach does not encompass the entire apoptotic landscape. Future studies should consider additional markers of apoptosis, such as autophagy indicators and oxidative stress regulators, to provide a more comprehensive understanding of the apoptotic processes at play in oocytes infected with BVDV.

## 5 Conclusion

In conclusion, this study demonstrates that the CP biotype of BVDV can induce apoptosis in oocytes, potentially compromising their quality. The findings underscore the importance of understanding the precise mechanisms through which BVDV triggers apoptosis to develop targeted interventions. Further comprehensive investigations are warranted to unravel the intricate molecular pathways involved in BVDV‐induced apoptosis and its impact on oocyte quality. Such knowledge will contribute to the development of effective strategies for mitigating the adverse effects of BVDV infection on reproductive outcomes.

## Author Contributions

Massoud Talebkhan Garoussi: Conceptualisation, Data curation, Formal analysis, Funding acquisition, Investigation, Methodology, Project administration, Resources, Supervision, Validation, Visualisation, Writing original draft, Writing—review & editing. Amirmahdi Roshanzamir: Conceptualisation, Formal analysis, Investigation, Methodology, Resources, Software, Validation, Visualisation, Writing—original draft, Writing—review & editing. Jalil Mehrzad: Conceptualisation, Formal analysis, Funding acquisition, Investigation, Methodology, Project administration, Resources, Software, Supervision, Validation, Visualisation, Writing—original draft, Writing—review & editing.

## Ethics Statement

The authors confirm that the ethical policies of the journal, as noted on the journal's author guidelines page, have been adhered to and the appropriate ethical review committee approval has been received.

## Conflicts of Interest

The authors declare no conflicts of interest.

### Peer Review

The peer review history for this article is available at https://publons.com/publon/10.1002/vms3.70216.

## Data Availability

The data supporting this study's findings are available on request from the corresponding author.
